# Supplementary material for: Centroid of the bacterial growth curves: a metric to assess phage efficiency
Source: Commun Biol. 2024 May 31;7:673. doi: 10.1038/s42003-024-06379-z (PMC11143336; doi:10.1038/s42003-024-06379-z)
Supplement: Supplementary file 2 — Supplementary Information [file 42003_2024_6379_MOESM2_ESM.pdf]

Centroid of the bacterial growth curves: A metric to assess phage efficiency

Nava Hosseini[1,2,*], Mahdi Chehreghani[3], Sylvain Moineau[1,2,4,5] and Steve J. Charette[1,2,6,*]

1. Institut de Biologie Intégrative et des Systèmes (IBIS), Pavillon Charles-Eugène-Marchand, Université Laval, Quebec City, QC, G1V 0A6, Canada.
2. Département de biochimie, de microbiologie et de bio-informatique, Faculté des sciences et de génie, Université Laval, Quebec City, QC, G1V 0A6, Canada.
3. Department of Mechanical Engineering, McGill University, Montreal, QC, H3A 0C3, Canada.
4. Groupe de Recherche en Écologie Buccale (GREB), Faculté de médecine dentaire, Université Laval, Quebec City, QC, G1V 0A6, Canada.
5. Félix d'Hérelle Reference Center for Bacterial Viruses, Université Laval, Quebec City, QC, G1V 0A6, Canada.
6. Centre de Recherche de l'Institut Universitaire de Cardiologie et de Pneumologie de Québec (IUCPQ), Quebec City, QC, G1V 4G5, Canada.

*Correspondance: Institut de Biologie Intégrative et des Systèmes (IBIS), Pavillon Charles-Eugène-Marchand, Université Laval, Quebec City, QC, G1V 0A6, Canada nava.hosseini.1@ulaval.ca, steve.charette@bcm.ulaval.ca.

Supplementary Table 1. Bacteriophages used in cocktail combinations.

Bacteriophage	Phage Morphology	GenBank Accession Number	Origin	Ref.
44RR2.8t.2	Myovirus	KY290948	Water, Ontario, Canada	FHRC[a]
65.2	Myovirus	KY290955	River, Saône-et-Loire, France	FHRC
MQM1	Podophage	OQ628262	Fish mucus, Quebec, Canada	[1]
Riv-10	Myovirus	KY290957	River, Quebec, Canada	[2]
SW69-9	Myovirus	KY290958	Source, Quebec, Canada	[2]
SW69-9.BK93	Myovirus	-	Derivative of phage SW69-9	-

[a]FHRC: Felix d'Herelle Reference Center for Bacterial Viruses (www.phage.ulaval.ca).

Supplementary Table 2. *Aeromonas salmonicida* subsp. *salmonicida* strains used in this study.

Bacterial Strain	Other Names	Origin	*AsaGEI*[a]	Gene Cluster[b]	TTSS[c]	A-layer	Antibiotic Resistance Genes[d]	Ref.
01-B516	I, or B	Quebec, Canada	*2a*	No	Yes	Yes	None	[3]
2004-05 MF26	II	New-Brunswick, Canada	*2a*	No	Yes	Yes	*sul1, sul2, floR, tetA*	[4]
01-B526	A	Quebec, Canada	*1a*	Yes	Yes	Yes	None	[5]
M22710-11	C	Quebec, Canada	*2a*	No	Yes	Yes	None	[6]
SHY18-4069	D	Quebec, Canada	-	No	Yes	Yes	None	[7]

a: Genomic islands (prophage-derived) found in *Aeromonas salmonicida* subsp. *salmonicida.*
b: The gene cluster correspond to genes ASA_2927 to ASA_2933 in strain A449[7].
c: Type three secretion system (TTSS).
d: Only known antibiotic resistance genes to tetracycline, florfenicol, chloramphenicol, and sulfonamide are indicated.

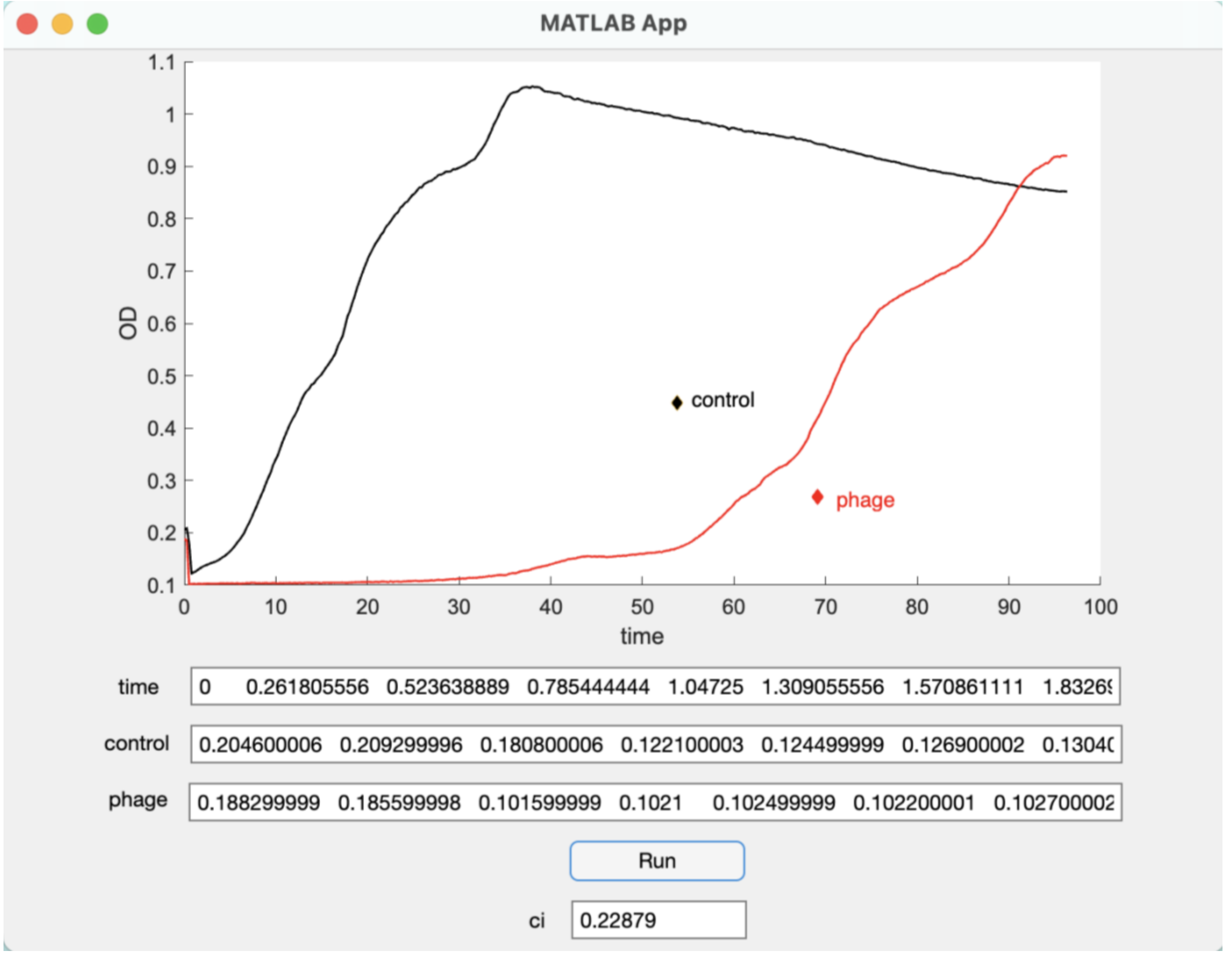

Supplementary Figure 1. An overview of the Centroid Index Calculator software. This software is designed with MATLAB R2023a. In order to calculate the centroid index (CI) of a desired phage-treated bacterium, the OD data of the phage-treated bacteria and the control bacteria are entered in the designated box ("phage" & "control" boxes, respectively). By running the application, the data and the centroid locations will be plotted, and the value of CI will be calculated by the software.

Supplementary Notes 1.

Installation of Centroid Index Calculator software:

Please download the Installer from https://doi.org/10.5281/zenodo.11137800, or https://github.com/navahs/Centroid-Index-Calculator.git.

Currently, the Centroid_Index_Calculator is available for the Windows and MacOS.

1. Installation instructions for MacOS

After downloading Centroid_Index_Calculator_Installer, during the installation, one may see this error message:

> "Centroid_Index_Calculator_Installer" cannot be open because the developer cannot be verified".

It might be necessary to refer to MacOS setting to make changes to allow the app to be installed, as follows:

Security & privacy>General>App store & identified developer>Open anyway.

Then, the installer will pop up on the screen.

The installation steps include:

DESTINATION>MATLAB RUNTIME>CONFIRMATION

This way, the "MATLAB Runtime" will be automatically downloaded on the system.

To open the software, please refer to:

Applications>Centroid_Index_Calculator folder>Application>Centroid_Index_Calculator

After some time, the "MATLAB app" will appear on the screen.

2. Installation instructions for Windows

The installation of the software is more straightforward for Windows. The same "warning" notification might appear during the software installation due to security and privacy which is easily solvable.

Notes:

1. This software is developed to process an Excel file data, in which the data are (by default) spaced by "tab". Therefore, the only step for the user is to copy/paste data from Excel file to the appropriate boxes in the software.

2. If one chose to enter the data manually, regardless of whether the data points are separated by "comma" or "tabs", it is necessary to be consistent for all data. For example, the software will not be able to analyze the data and calculate the CI if the data for "time" are separated by "comma" and data for OD are separated by "tab".
3. The software does not work for data with decimal points represented as "comma" instead of "period". For example, the data must be 0.2 and not 0,2. This problem might appear if the Excel file is in a language other than English.
4. Once the calculations are done for a series of data, it is possible to delete the calculated value of CI in the associated box and then enter a new data set.

Supplementary References

1 Hosseini, N. *et al.* MQM1, a bacteriophage infecting strains of *Aeromonas salmonicida* subspecies *salmonicida* carrying Prophage 3. *Virus Res.* 334, 199165 (2023). https://doi.org:10.1016/j.virusres.2023.199165

2 Vincent, A. T. *et al.* Characterization and diversity of phages infecting *Aeromonas salmonicida* subsp. *salmonicida*. *Sci. Rep.* 7, 7054 (2017). https://doi.org:10.1038/s41598-017-07401-7

3 Daher, R. K. *et al.* Alteration of virulence factors and rearrangement of pAsa5 plasmid caused by the growth of *Aeromonas salmonicida* in stressful conditions. *Vet. Microbiol.* 152, 353-360 (2011). https://doi.org:10.1016/j.vetmic.2011.04.034

4 Vincent, A. T. *et al.* Detection of variants of the pRAS3, pAB5S9, and pSN254 plasmids in Aeromonas salmonicida subsp. salmonicida: multidrug resistance, interspecies exchanges, and plasmid reshaping. *Antimicrob. Agents Chemother* 58, 7367-7374 (2014). https://doi.org:10.1128/AAC.03730-14

5 Dautremepuits, C., Fortier, M., Croisetiere, S., Belhumeur, P. & Fournier, M. Modulation of juvenile brook trout (*Salvelinus fontinalis*) cellular immune system after *Aeromonas salmonicida* challenge. *Vet. Immunol. Immunopathol.* 110, 27-36 (2006). https://doi.org:10.1016/j.vetimm.2005.09.008

6 Emond-Rheault, J. G. *et al.* Variants of a genomic island in *Aeromonas salmonicida* subsp. *salmonicida* link isolates with their geographical origins. *Vet. Microbiol.* 175, 68-76 (2015). https://doi.org:10.1016/j.vetmic.2014.11.014

7 Marcoux, P. E. *et al.* Systematic analysis of the stress-induced genomic instability of type three secretion system in *Aeromonas salmonicida* subsp. *salmonicida*. *Microorganisms* 9, 85 (2020). https://doi.org:10.3390/microorganisms9010085
