# Supplementary material for: Centroid of the bacterial growth curves: a metric to assess phage efficiency
Source: Commun Biol. 2024 May 31;7:673. doi: 10.1038/s42003-024-06379-z (PMC11143336; doi:10.1038/s42003-024-06379-z)
Supplement: Supplementary file 3 — Description of Additional Supplementary Files [file 42003_2024_6379_MOESM3_ESM.pdf]

Description of Additional Supplementary Files

File name: Supplementary Data 1
Description: OD data & Formula sheets for VI & CI calculations
